# Role of crescents for lupus nephritis in clinical, pathological and prognosis: a single-center retrospective cohort study

**DOI:** 10.1186/s40001-023-01022-9

**Published:** 2023-02-02

**Authors:** Sishi Lin, Ji Zhang, Bo Chen, Duo Li, Yan Liang, Ya Hu, Xueting Liu, Yongheng Bai, Chaosheng Chen

**Affiliations:** 1 Department of Nephrology, The First Affiliated Hospital of Wenzhou Medical University, Wenzhou, 325000 Zhejiang People’s Republic of China; 2 Institute of Chronic Kidney Disease, Wenzhou Medical University, Wenzhou, 325000 Zhejiang People’s Republic of China; 3 Key Laboratory of Diagnosis and Treatment of Severe Hepato-Pancreatic Diseases of Zhejiang Province, The First Affiliated Hospital of Wenzhou Medical University, Wenzhou, China

**Keywords:** Lupus nephritis, Crescent, Renal pathology, Outcome

## Abstract

**Background:**

Referring to the International Society of Nephrology/Renal Pathology Society (ISN/RPS) 2018 pathological classification, we aim to reveal the significance of cellular/fibrocellular crescents in lupus nephritis (LN) patients.

**Methods:**

Patients with LN proven by renal biopsy at the First Affiliated Hospital of Wenzhou Medical University from December 2001 to November 2017 were identified, and eligible cases were divided into two groups according to the presence or absence of cellular/fibrocellular crescents in renal biopsy tissues.

**Results:**

A total of 401 LN patients were identified from our follow-up database, and 296 eligible LN patients were enrolled in the study. Of these patients, 146 patients in the group without cellular/fibrocellular crescents (non-crescent group) and 150 patients in the group with cellular/fibrocellular crescents (Crescent group). The median follow-up time of patients was 47 months, and a total of 54 patients progressed to the composite endpoint. Crescent group had higher serum creatinine, lower serum albumin, higher systemic lupus erythematosus (SLE) disease activity index, and higher activity index of renal tissue. The interaction between cellular/fibrocellular crescents and proteinuria at baseline was associated with the prognostic risk of LN (*P* = 0.006). In the group with proteinuria < 3.5 g/24 h, the prognosis of crescent group was significantly worse than of non-crescent group (*P* < 0.001), while in the group with proteinuria ≥ 3.5 g/24 h, there was no significant relationship between crescents and prognosis (*p* = 0.452). By multivariable Cox hazard analysis, positive anti-dsDNA, chronic index of renal biopsy tissue, cellular/fibrocellular crescents and its interaction with 24 h proteinuria were independent risk factors for poor prognosis of LN.

**Conclusions:**

LN patients with cellular/fibrocellular crescents had more severe and active disease features, and cellular/fibrocellular crescents is a risk factor for poor prognosis of LN. There was an interaction between cellular/fibrocellular crescents and proteinuria in predicting poor prognosis, and among patients with low levels of proteinuria at the time of renal biopsy, those with crescents had a worse long-term prognosis than those without crescents.

**Supplementary Information:**

The online version contains supplementary material available at 10.1186/s40001-023-01022-9.

## Background

Lupus nephritis (LN) is a common manifestation of systemic lupus erythematosus (SLE) and an important cause of disease progression and death. Studies have shown that 5 ~ 25% of proliferative LN patients die directly from kidney disease within 5 years of onset, and 10 ~ 30% of LN patients progress to end-stage renal disease (ESRD) [1]. The diverse renal pathology of LN results in individual differences in response to treatment and prognosis, the relationship between crescents and clinical manifestation and prognosis of LN remain ambiguous. Zhang et al. showed that although a higher proportion of crescents increased the risk of adverse events, it was not associated with the long-term outcome of LN [2]; Kornwipa et al. found that cellular crescent was negatively associated with achieving renal remission following immunosuppressive treatment [3], while Cai et al. found that proliferative LN patients with crescents > 25% had a poor short- and long-term renal prognosis [4]. In this study, based on the ISN/RPS 2018 pathological classification, we retrospectively investigated the clinical and pathological characteristics of LN patients with or without cellular/fibrocellular crescents in renal tissue from our center, and analyzed their relationship with the prognosis of LN.

## Methods

### Aim

This study aimed to investigate the clinical and pathological characteristics of LN patients with or without cellular/fibrocellular crescents in renal tissue, and to analyze their relationship with the prognosis of LN.

### Study design

This study was a retrospective cohort study using data from the follow-up database of the First Affiliated Hospital of Wenzhou Medical University.

### Study population

**Fig. 1:**
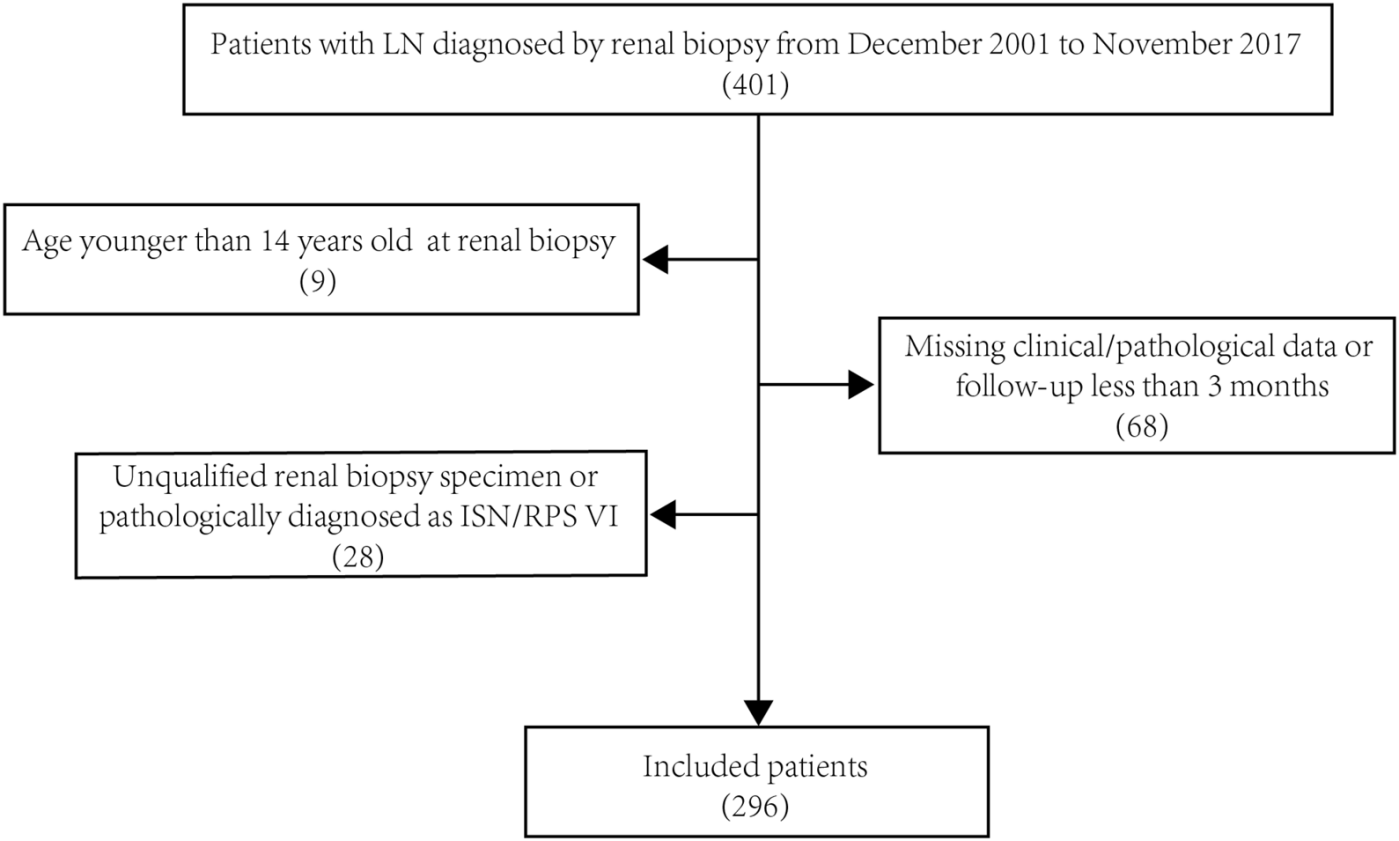
Selection process for eligible patients

A total of 401 biopsy proven LN patients at the First Affiliated Hospital of Wenzhou Medical University from December 2001 to November 2017 were identified. The diagnostic of SLE was based on EULAR/ACR 2019 classification criteria [6], and the histological classification of LN was based on the ISN/RPS 2018 classification criteria [5]. Exclusion criteria: (1) the clinical/pathological data were missing or the follow-up time < 3 months; (2) the quality of renal biopsy specimens was unqualified or pathologically diagnosed as ISN/RPS class VI; and (3) age < 14 years at the time of renal biopsy. Finally, 296 cases were included (Fig. 1). Patients were divided into 2 groups according to the presence or absence of cellular/fibrocellular crescents (the ‘crescent’ mentioned later refers to the cellular/fibrocellular crescent) in renal tissue: non-crescent group (without crescents) and Crescent group (with crescents).

### Histological classification

Biopsy specimens were embedded in paraffin and sectioned, and stained with HE, PAS, Masson and direct immunofluorescence. All the biopsy specimens were processed by standard light and immunofluorescence, and 80–90% were examined by electron microscopy. Two pathologists each reviewed and reclassified the renal pathology data according to the ISN/RPS 2018 classification criteria without knowing of the patient's clinical data, and if there was a discrepancy between the two pathologists, biopsies were reviewed until consensus was reached.

**Fig. 2:**
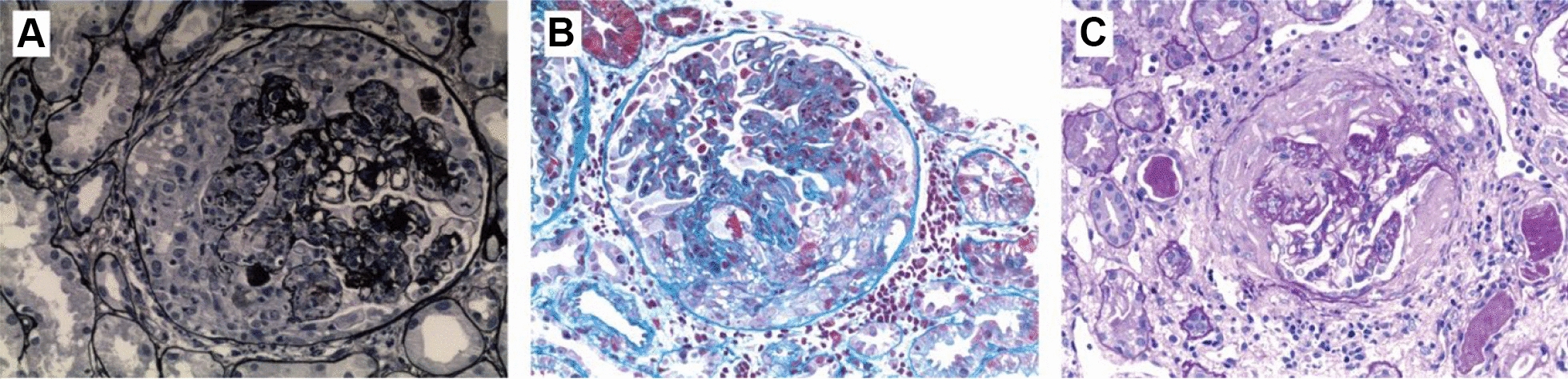
Crescent formation in pathological specimens of lupus nephritis. **A** Formation of large cellular crescent in the left side of glomerulus, and severe proliferation of cells in the capillaries in the right side of glomerulus with polymorphonuclear cells retention (PASM × 400). **B** Formation of large fibrocellular crescent in the lower right side of the glomerulus, severe proliferation of cells in the segmental capillaries in the upper right side of the glomerulus, and fibrinoid necrosis in the segmental capillary loop in the lower right side of the glomerulus (MASSON × 400). **C** Formation of large fibrous crescent in glomerulus, focal segmental glomerulosclerosis in the upper left side of glomerulus (PAS × 400)

The crescent is defined as a lesion consisting of extracapillary hypercellularity, composed of a variable mixture of cells, which may contain fibrin and fibrous matrix and should involve ≥ 10% of the circumference of Bowman's capsule; cellular crescent defined as crescent composed of > 75% cells and fibrin and < 25% fibrous matrix; fibrocellular crescent defined as composed of 25 ~ 75% cells and fibrin and the remainder fibrous matrix; fibrous crescent defined as composed of > 75% fibrous matrix and < 25% cells and fibrin [5] (Fig. 2). In addition, the activity index (AI) and chronic index (CI) of renal tissue were scored semi-quantitatively according to the proposed modified NIH lupus nephritis activity and chronic scoring system revised by ISN/RPS 2018 classification criteria [5].

### Clinical data

Baseline information were collected from the hospital information system (patients with repeated renal biopsies took the first renal biopsy as the baseline), including: age, sex, height, weight, body mass index (BMI) [weight (kg)/height (kg)^2^], blood pressure, mean arterial pressure (MBP) [diastolic + 1/3 (systolic–diastolic)], SLE disease activity index (SLEDAI) [7]; simultaneous collection of baseline and follow-up data, including: serum creatinine, estimated glomerular filtration rate (eGFR) [calculated by the 2021 CKD–EPI formula [8]], serum albumin, white blood cells, hemoglobin, platelets, 24 h proteinuria, urine white blood cells (WBC), urine red blood cells (RBC), urinary albumin/creatinine ratio (ACR), serum antinuclear antibody, anti-dsDNA, anti-Sm, anti-u1RNP, anti-SSA, anti-SSB antibody, lupus anticoagulant ratio, complement C3, C4, and major treatment regimens received at baseline and during the first 24 months of follow-up (glucocorticoid and cyclophosphamide therapy).

### Renal survival

The composite endpoint was defined as a decrease in eGFR of > 30%, ESRD or the need of dialysis and/or kidney transplantation, and death. The follow-up time was the time of the first endpoint event.

### Statistical analysis

Continuous variables with normal distribution were described by means ± standard deviation, and *t* test was used for comparison between groups. The continuous variables with non-normal distribution were described by median [quartile], and Wilcoxon rank sum test was used for comparison between groups. The classified variables were described by the number of cases (%), and the comparison between groups was described by χ2 or Fisher exact test. Kaplan–Meier curve (log-ranch test) was performed to demonstrate the different survival of poor outcomes of LN patients. Linear mixed model was built to display the different change of eGFR over time. Cox regression models were constructed to analyze the effects of clinicopathological characteristics on poor outcomes, and the results were expressed as hazard ratio (HR) and corresponding 95% confidence interval (CI). Interactions between variables were evaluated and a multivariate Cox regression model was constructed and optimized using forward–backward stepwise methods. The receiver operating characteristic (ROC) analysis was used to assess the performance of the optimal Cox regression model. A two-sided p value of < 0.05 was considered statistically significant. The R software (version 4.1.2) [9] and R packages (such as ggplot2 [10], Raincloud plot [11], Tableone [12]) to perform the analyses and plots.

## Results

### Analysis of baseline data

A total of 296 LN patients were enrolled in this study. The age at the time of renal biopsy ranged from 14 to 64 years, with an average age of 31.93 years, and male-to-female ratio was 0.14:1. According to the ISN/RPS 2018 LN classification criteria, there were 1 case of class I (0.3%), 6 cases of class II (2.0%), 82 cases of class III lesions (including 54 cases of class III + V and 28 cases of class III) (27.7%), 160 cases of class IV lesions (including 57 cases of class IV + V and 103 cases of class IV) (54.1%) and 47 cases of class V (15.9%). The median follow-up time was 47 [23.75, 83] months, and 54 patients (18.2%) reached composite endpoint event (Table 1).

**Table 1:** Baseline clinical and pathological data of lupus nephritis patients

	Overall
Number of patients	296
Age (year) (mean (S.D.))	31.93 (10.51)
Gender = male, *n* (%)	42 (14.2)
ISN/RPS class, *n* (%)	
Class I	1 (0.3)
Class II	6 (2.0)
Class III	82 (27.7)
Class IV	160 (54.1)
Class V	47 (15.9)
Follow-up month (median [IQR])	47 [23.75, 83]
Composite events, n (%)	54 (18.2)

*ISN/RPS* international society of nephrology/renal pathology society,* SD*, standard deviation, *IQR* interquartile range

### Comparison of baseline data between groups

There were no significant differences in demographic characteristics between the two groups. The median follow-up time of non-crescent group was longer than that of crescent group (57 months vs. 38.5 months, *P* < 0.001). Compared with non-crescent group, MBP (*P* < 0.001), serum creatinine (*P* < 0.001), WBC count (*P* = 0.019), 24-h proteinuria (*P* < 0.001), urinary ACR (*P* < 0.001), urine WBC count (*P* < 0.001), urine RBC count (*P* < 0.001), proportion of positive anti-dsDNA antibodies (*P* < 0.001), and SLEDA index (*P* < 0.001) were higher, while eGFR (*P* < 0.001), serum albumin (*P* = 0.001), hemoglobin (*P* < 0.001), complement C3 (*P* = 0.007), complement C4 (*P* = 0.015), and lupus anticoagulant ratio (*P* < 0.001) were lower in crescent group. The rest of the clinical indexes were not significantly different (Table 2).

**Table 2:** Baseline clinical characteristics of LN patients stratified by crescent

	Non-crescent	Crescent	*P* value
Number of patients	146	150	
Gender = male, *n* (%)	19 (13.0)	23 (15.3)	0.619
Age (year) (mean (S.D.))	31.93 (10.16)	31.93 (10.86)	0.997
BMI (mean (S.D.))	21.69 (2.93)	21.95 (2.86)	0.429
MBP (mmHg) (mean (S.D.))	97.98 (17.01)	105.38 (16.15)	< 0.001
Serum creatinine (μmol/L) (median [IQR])	52.38 [46.21, 63.75]	69.09 [55.62, 90.63]	< 0.001
eGFR (median [IQR])	122.88 [111.63, 131.53]	105.91 [76.42, 125.30]	< 0.001
Serum albumin (g/L) (median [IQR])	28.30 [23.13, 33.74]	25.43 [21.94, 28.81]	0.001
WBC count (10^9/L) (median [IQR])	6.80 [5.12, 8.86]	7.87 [6.16, 9.67]	0.019
Hemoglobin (g/L) (median [IQR])	110.02 [96.38, 121.15]	98.70 [84.72, 111.08]	< 0.001
Platelet (10^9/L) (median [IQR])	183.50 [159.33, 233.25]	181.50 [142.00, 226.67]	0.413
Proteinuria (g/24-h) (median [IQR])	2.08 [0.96, 4.10]	3.52 [2.02, 5.91]	< 0.001
Urinary albumin/creatinine ratio (mg/μmol) (median [IQR])	0.22 [0.11, 0.44]	0.40 [0.22, 0.61]	< 0.001
Urine WBC (/HP) (median [IQR])	2.08 [1.12, 5.50]	6.51 [3.60, 11.35]	< 0.001
Urine RBC (/HP) (median [IQR])	24.83 [8.22, 38.20]	37.90 [19.60, 72.14]	< 0.001
C3 (g/L) (median [IQR])	0.48 [0.36, 0.69]	0.43 [0.35, 0.54]	0.007
C4 (g/L) (median [IQR])	0.10 [0.05, 0.14]	0.07 [0.05, 0.11]	0.015
Positive ANA, *n* (%)	91 (62.3)	109 (72.7)	0.063
Positive anti-cardiolipin, *n* (%)	1 (0.7)	3 (2.0)	0.623
Positive anti-dsDNA, *n* (%)	51 (34.9)	88 (58.7)	< 0.001
Positive anti-Ro52, *n* (%)	17 (11.6)	30 (20.0)	0.057
Positive anti-Sm, *n* (%)	33 (22.6)	38 (25.3)	0.590
Positive anti-ssA, *n* (%)	79 (54.1)	80 (53.3)	0.908
Positive anti-ssB, *n* (%)	28 (19.2)	22 (14.7)	0.353
Positive anti-u1RNP, *n* (%)	53 (36.3)	62 (41.3)	0.405
Lupus anticoagulant ratio (median [IQR])	1.10 [0.99, 1.25]	1.00 [0.84, 1.15]	< 0.001
SLEDAI(median [IQR])	16.00 [11.00, 19.00]	18.00 [15.00, 22.00]	< 0.001
Follow-up month (median [IQR])	57.00 [30.50, 96.50]	38.50 [19.00, 66.75]	< 0.001

*BMI* body mass index, *MBP* mean blood pressure, *eGFR* estimated glomerular filtration rate, *WBC* white blood cells, *RBC* red blood cells, *C3* complement factor 3, *C4* complement factor 4, *ANA* antinuclear antibodies, *dS.D.NA* double-stranded DNA, *ssA* Sjögren’s-syndrome-related antigen A; ssB, Sjögren’s-syndrome-related antigen B; u1RNP, U1 ribonucleoprotein; SLEDAI, systemic lupus erythematosus disease activity index, *S.D*, standard deviation, *IQR*, interquartile range

**Fig. 3:**
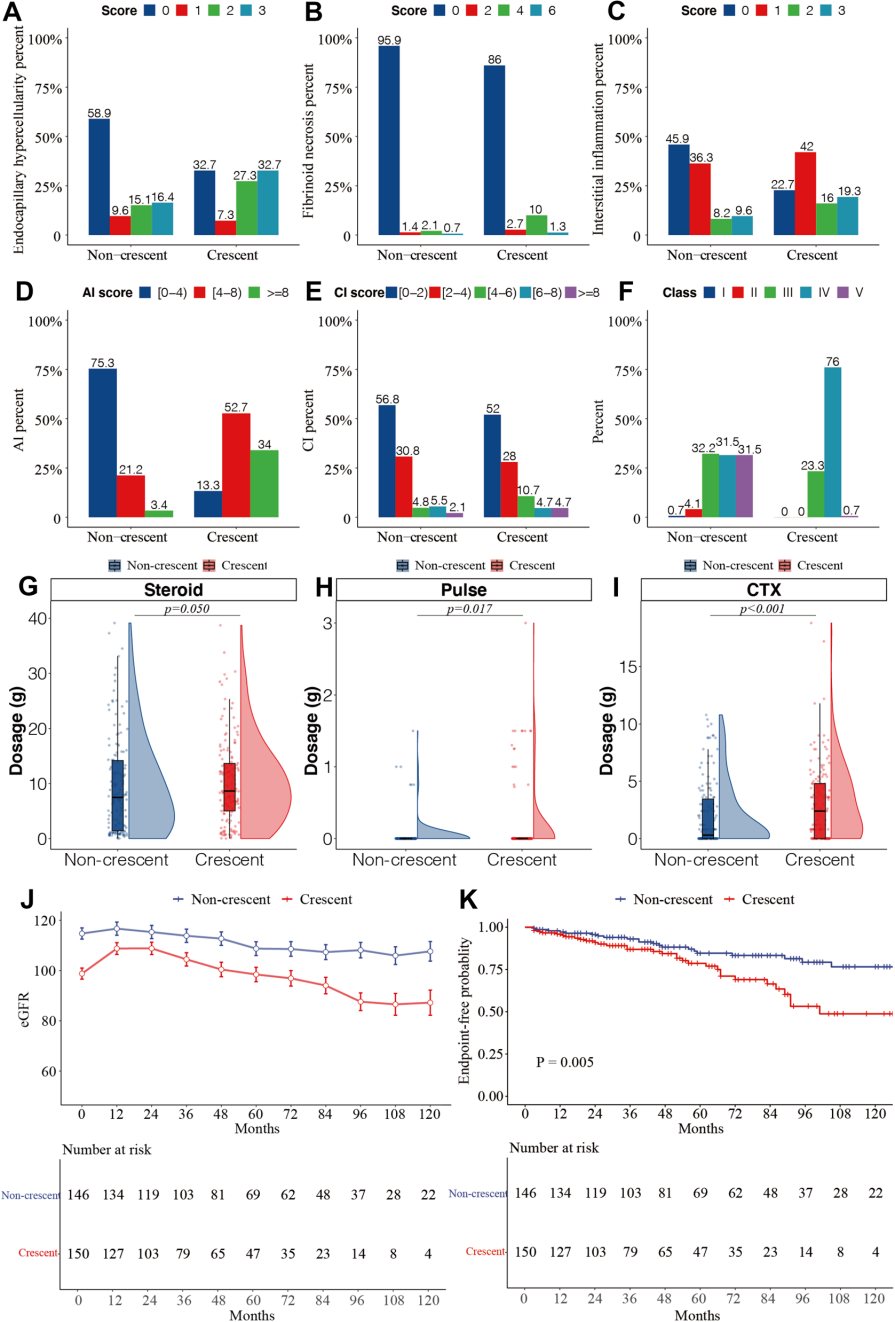
Comparison of clinical, pathological and prognosis between patients with or without crescents. **a**–**f** According to ISN/RPS 2018 pathological classification, the AI and CI of renal tissue were evaluated, and the histological classification were classified, to compare the pathological manifestations between patients with or without crescents; **g**–**i**: comparison of the main treatment regimens in the first 24 months of follow-up between patients with or without crescents; **j** estimated marginal means of eGFR (*y*-axis) for the non-crescents group and crescents group across timepoints (*x*-axis). Error bars indicate standard error; **k** comparison of prognosis between patients with or without crescents (Kaplan–Meier curve), *p* = 0.011 (log-rank test), the end points is eGFR 30% decrease of the baseline or reaching end-stage renal disease or need for dialysis or renal transplantation or death. *AI*, activity NIH index, *CI*, chronicity NIH index, *Pulse* methylprednisolone pulse; *CTX* cyclophosphamide, *eGFR* estimated glomerular filtration rate

The most common histological classification in non-crescent group was class III (32.2%), while the proportion of class IV (76.0%) in crescent group was significantly higher than that of other pathological classifications. According to proposed modified NIH lupus nephritis activity and chronic scoring system, the scores of endocapillary hypercellularity (*P* < 0.001), interstitial inflammation (*P* < 0.001) and fibrinoid necrosis (P = 0.007) were higher in crescent group. AI score was higher in crescent group (1 [0, 3] vs. 6 [4.25,8], *P* < 0.001), with no significant difference in CI score (1 [0,2.75] vs. 1 [0,3], *P* = 0.153) (Fig. 3A–F).

### Comparison of therapeutic regimen and clinical outcome between groups

Comparing the major treatment regimens received at baseline and during the first 24 months of follow-up between the two groups, total dose of glucocorticoid (*P* = 0.050), proportion (*P* = 0.028) and total dose (*P* = 0.017) of methylprednisolone pulse therapy, and total dose of cyclophosphamide (*P* < 0.001) were higher in the crescent group than that in the non-crescent group (Fig. 3G–I). In addition, the drugs used to maintain remission during maintenance remission period were compared between the two groups. The results showed that there was no significant difference in the proportion of hydroxychloroquine, azathioprine, calcineurin inhibitors and RA system inhibitors between the two groups (Additional file 1).

The changes of eGFR over time in both groups are shown in Fig. 3J. As the disease progressed, eGFR in both groups tended to decrease, and there was a significant interaction between crescents and time in the detection of eGFR (*P* = 0.035). However, eGFR in crescent group gradually increased during the first 24 months of follow-up and decreased thereafter.

There was no significant difference in the proportion of endpoint events between non-crescent group and crescent group (14.4% vs. 22.0%, *P* = 0.099), but the median follow-up time of crescent group was shorter than that of non-crescent group (57 months vs. 38.5 months, *P* < 0.001). Long-term prognostic assessment of LN patients who were followed up for > 3 months revealed that the overall long-term prognosis was worse in crescent group (*P* = 0.005) (Fig. 3K).

### Interaction between crescents and clinical parameters and its effects on LN

Analysis of the interaction between crescents and clinical characters showed a significant interaction between crescents and proteinuria levels at baseline, which was related to the outcome of LN (*P* = 0.006) (Additional file 2). Meanwhile, patients with different levels of proteinuria were treated differently (Additional file 3). Therefore, 24-h proteinuria was further divided into two levels: < 3.5 g/24 h and ≥ 3.5 g/24 h. Further stratified analysis showed that there was a significant correlation between crescents and prognosis of LN in the group with proteinuria < 3.5 g/24 h at the time of renal biopsy, and the prognosis of LN in the group with crescents was significantly worse than that in the group without crescents (*P* < 0.001), but there was no significant correlation between crescents and prognosis in the groups with proteinuria ≥ 3.5 g/24 h (Table 3).

**Table 3:** Stratified associations between Crescent and prognosis of LN by proteinuria

	Crescent		*P* value	*P* value for interaction^b^
	Non-crescent	Crescent^a^		
Proteinuria				
< 3.5 g/24 h	1	2.68(1.64–4.38)	< 0.001	0.006
≥ 3.5 g/24 h	1	0.77(0.39–1.52)	0.452	

^a^Hazard ratio (95% confidence interval); ^b^ P value for interaction test: two-way interaction of proteinuria (< 3.5 g/24 h vs. ≥ 3.5 g/24 h) and crescent groups (non-crescent vs. crescent) on the prognosis of lupus nephritis

**Fig. 4:**
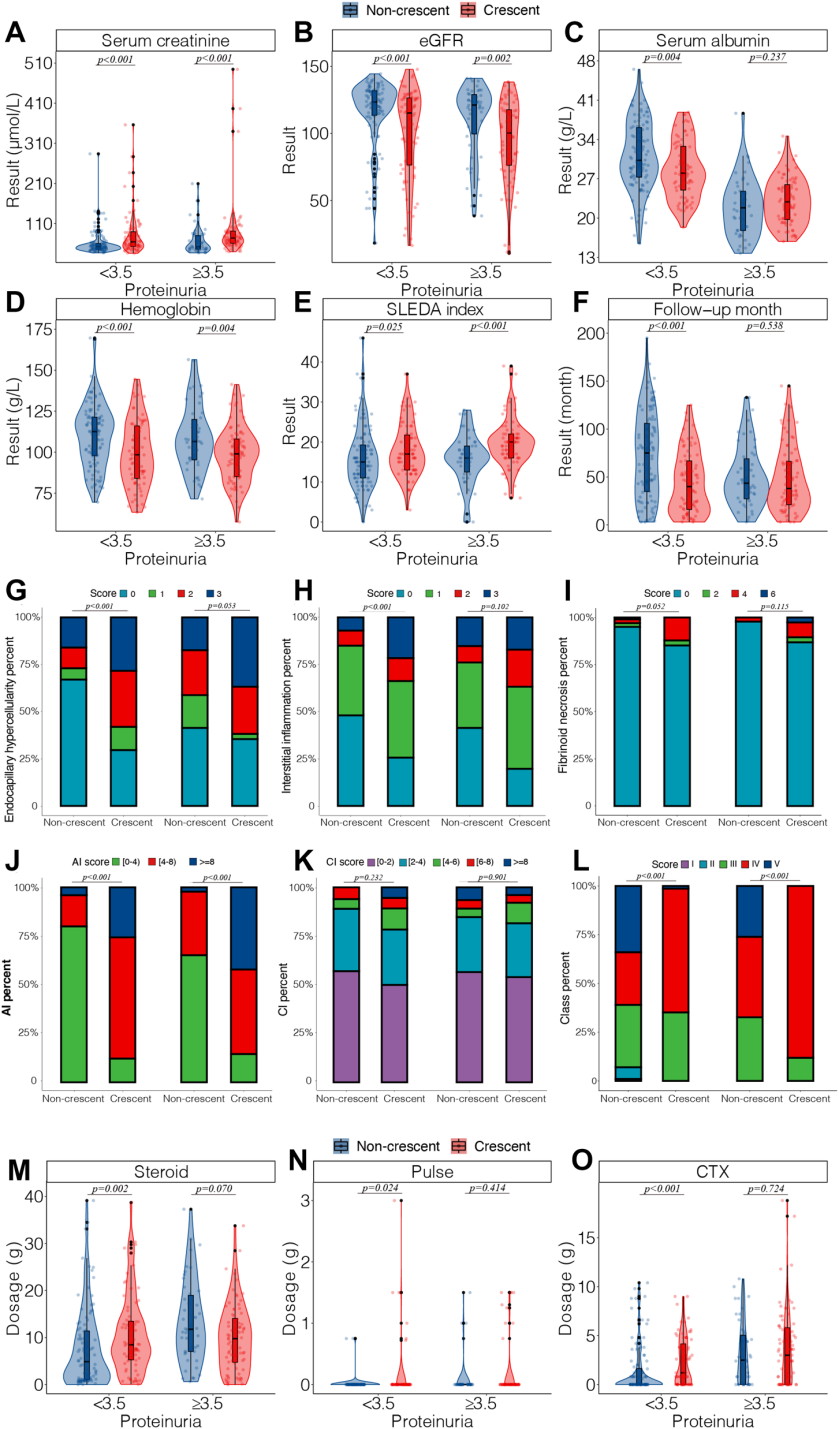
Comparison of clinical and pathological characteristics between patients with or without crescents, grouped by 24 h proteinuria. **a**–**f** Comparison of clinical characteristics between patients with or without crescents, grouped by 24 h proteinuria; **g**–**l** according to ISN/RPS 2018 pathological classification, the AI and CI of renal tissue were evaluated and histologically classified, to compare the pathological characteristics between patients with or without crescents, grouped by 24 h proteinuria; **m**–**o** comparison of the main treatment regimens in the first 24 months of follow-up between patients with or without crescents, grouped by 24 h proteinuria; *eGFR* estimated glomerular filtration rate, *SLEDAI* systemic lupus erythematosus disease activity index, *AI*, activity NIH index, *CI*, chronicity NIH index; Pulse, methylprednisolone pulse, *CTX* cyclophosphamide. *p < 0.05 compared with non-crescent group; **p < 0.01 compared with non-crescent group

In both groups with different proteinuria levels, serum creatinine, urine WBC count, urine RBC count, and SLEDAI were higher, while eGFR and hemoglobin were lower in the group with crescents. In the group with proteinuria < 3.5 g/24 h, the proportion of positive anti-dsDNA antibodies (*P* < 0.001) and antinuclear antibodies (*P* = 0.040) was higher, while serum albumin (*P* = 0.004) was lower, and the median follow-up time was shorter (*P* < 0.001) in the group with crescents, but in the group with proteinuria ≥ 3.5 g/24 h, there was no significant difference between the groups with or without crescent (Fig. 4A–F). In terms of pathological manifestations, in the group of proteinuria < 3.5 g/24 h, the most common histological classification was class V (34.0%) in the group without crescent and class IV (63.5%) in the group with crescent, and compared to the group without crescent, the scores of endocapillary hypercellularity (*P* < 0.001), interstitial inflammation (*P* < 0.001), fibrinoid necrosis (*P* = 0.052) and AI scores (*P* < 0.001) were higher in the crescent group. In the proteinuria ≥ 3.5 g/24 h group, the most common histological classification was class IV (41.3%; 88.2%), with a higher AI score (*P* < 0.001) in the group with crescent, while the remaining pathological features were not significantly different between the groups with or without crescent (Fig. 4G–L). Comparing treatment regiments at baseline and during the first 2 years of follow-up, in the group with proteinuria < 3.5 g/24 h, the total dose of glucocorticoid (*P* = 0.002) and cyclophosphamide (*P* < 0.001) was higher in the group with crescent, and patients with crescent received a higher dose (*P* = 0.026) and proportion (*P* = 0.038) of methylprednisolone pulse therapy. In the proteinuria ≥ 3.5 g/24 h group, there was no significant difference in treatment between groups with or without crescent (Fig. 4M–O).

Subgroups were divided according to the presence or absence of crescents and levels of 24 h proteinuria. In non-crescent group, the total dose of glucocorticoid (*P* < 0.001), methylprednisolone pulse therapy (*P* = 0.055), cyclophosphamide (*P* < 0.001) at baseline and during the first 2 years of follow-up in the group with proteinuria ≥ 3.5 g/24 h were higher than those in the group with proteinuria < 3.5 g/24 h, and the median follow-up time was shorter (75.00 months vs. 43.50 months, *P* = 0.006). In crescent group, the proportion of class III and IV was higher in the group with proteinuria ≥ 3.5 g/24 h, but there was no significant difference in the treatment regimens and median follow-up time between the two groups with different levels of proteinuria (Additional files 4, 5, 6).

### Risk factors influencing the prognosis of LN patients

Univariate Cox regression model was constructed to analyze the relationship between clinical factors and the composite endpoint of LN (Table 4). The results showed that mean arterial pressure (1 mmHg: hazard ratio (HR) = 1.02, 95% confidence interval (CI) 1–1.03; *P* = 0.035), positive antinuclear antibody (*HR* = 2.24, 95% CI 1.41–3.55; *P* < 0.001), positive anti-dsDNA antibody (*HR* = 2.06, 95% CI 1.39–3.05, *P* < 0.001), AI score (with every one point increase: *HR* = 1.07, 95% CI 1.01–1.15; *P* = 0.034), CI score (with every one point increase: *HR* = 1.22, 95% CI 1.1–1.34, *P* < 0.001), crescent (crescent/non-crescent: HR = 1.74, 95% CI 1.18–2.57; *P* = 0.006), and total glucocorticoid dosage at baseline and during the first 24 months of follow-up (with every one gram: *HR* = 1.04, 95% CI 1.02–1.07; *P* = 0.001) were associated with the prognosis of LN patients.

**Table 4:** Univariate Cox regression models for the prediction of poor prognosis

Factors	Univariate		
	Hazard ratio	95% confidence interval	*P* value
Gender = male	1.46	0.65–3.24	0.356
Age (year)	1.02	1–1.05	0.078
BMI	1.03	0.94–1.13	0.550
MBP (mmHg)	1.02	1–1.03	0.035
Serum creatinine (μmol/L)	1	1–1.01	0.224
eGFR	0.99	0.98–1	0.062
Serum albumin (g/L)	1	0.96–1.04	0.874
WBC count (10^9/L)	1.02	0.93–1.11	0.679
Hemoglobin (g/L)	0.99	0.98–1	0.191
Platelet (10^9/L)	1	1–1	0.722
Proteinuria (g/24 h)	1.02	0.93–1.13	0.664
Urinary albumin/creatinine ratio (mg/μmol)	1.41	0.81–2.46	0.229
Urine WBC (/HP)	1.02	1–1.04	0.087
Urine RBC (/HP)	1	1–1	0.951
C3 (g/L)	0.82	0.25–2.7	0.739
C4 (g/L)	0.14	0–9.59	0.358
Positive ANA	2.24	1.41–3.55	< 0.001
Positive anti-dsDNA	2.06	1.39–3.05	< 0.001
SLEDAI	1.03	1–1.07	0.082
Modified NIH index			
AI	1.07	1.01–1.15	0.034
CI	1.22	1.1–1.34	< 0.001
Crescent			
Non-crescent	refer		
Crescent	1.74	1.18–2.57	0.006
Treatment			
Steroid dosage (g)	1.04	1.02–1.07	0.001
Proportion of pulse (g)	1.29	0.51–3.24	0.587
Pulse dosage (g)	0.95	0.47–1.92	0.878
CTX dosage (g)	1.05	0.97–1.14	0.229

*BMI* body mass index, *MBP* mean blood pressure, *eGFR* estimated glomerular filtration rate, *WBC* white blood cells, *RBC* red blood cells, *C3* complement factor 3, *C4* complement factor 4, *ANA* antinuclear antibodies, *dsDNA* double-stranded DNA, *SLEDAI* systemic lupus erythematosus disease activity index, *NIH* National Institutes of Health, *AI* activity NIH index, *CI* chronicity NIH index, *Pulse* methylprednisolone pulse, *CTX* cyclophosphamide

**Fig. 5:**
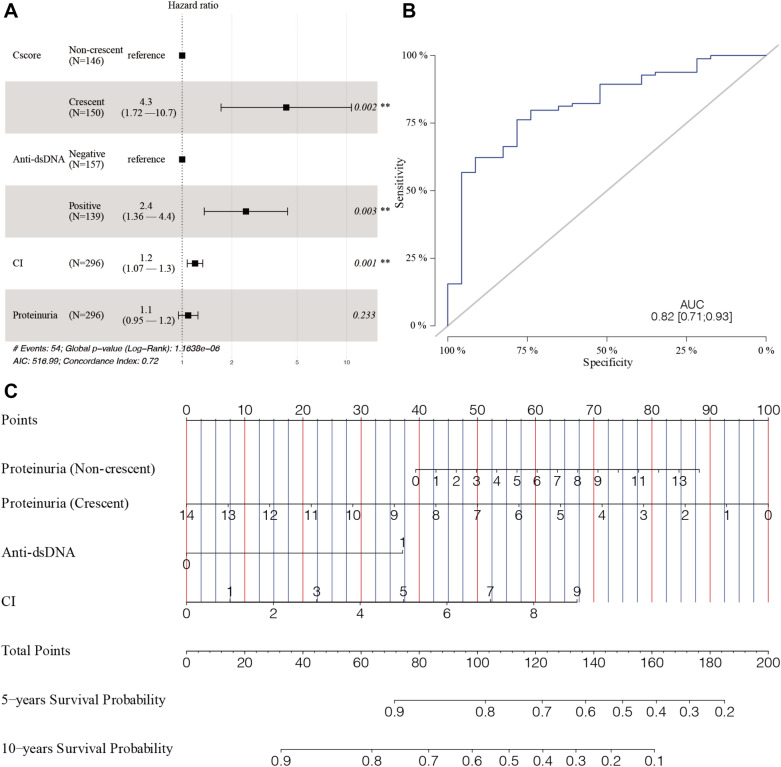
Construction and evaluation of the optimal multivariate Cox regression model for lupus nephritis. **A** Forest plot of composite endpoint. **B** Prediction performance of the optimal multivariate Cox regression model. A ROC plot by estimated 10-year survival probability of free from the composite endpoint. The area under the ROC curve (AUC) was 0.81 (95% CI 0.69–0.92). **C** A nomogram for the prediction of composite endpoint based on the optimal multivariate Cox regression model. The sum of these points, plotted on the “Total points” line, corresponds to the estimated probability of 5 years and 10 years free from the composite endpoint. Rows of proteinuria (non-crescent) and proteinuria (crescent) were the interaction items between proteinuria and crescents, and the numbers on the axis indicate the proteinuria level. *MBP* mean blood pressure, *dsDNA* double-stranded DNA, *SLEDAI*, systemic lupus erythematosus disease activity index, *CI* chronicity NIH index

Considering the interaction between crescent and 24 h proteinuria, multifactorial Cox regression model was constructed and optimized using forward–backward stepwise methods. The results showed that positive anti-dsDNA antibodies (95% CI 1.36–4.37; *P* = 0.003), CI score (95% CI 1.07–1.33; *P* = 0.001), crescent (95% CI 1.72–10.68; *P* = 0.002), and the interaction of crescent with 24-h proteinuria were independent risk factors for the composite endpoint of LN (Table 5, Fig. 5A).

**Table 5:** Multivariate Cox regression models for the prediction of poor prognosis

Factors	95% confidence interval	*P* value
Proteinuria (g/24-h)	0.95–1.25	0.233
Positive anti-dsDNA	1.36–4.37	0.003
CI	1.07–1.33	0.001
Crescent		
Non-crescent	refer	
Crescent	1.72–10.68	0.002
Interaction item		
Non-crescent: proteinuria	refer	
Crescent: proteinuria	0.63–0.95	0.015

Whole variables were included for constructing a full multivariate Cox regression model, and an optimal multivariate Cox regression model was selected using AIC with forward–backward steps. Because the model had interaction terms, the actual effect was not directly calculated. Only the coefficients and standard errors are displayed

*MBP* mean blood pressure, *dsDNA* double-stranded DNA, *SLEDAI* systemic lupus erythematosus disease activity index, *CI* chronicity NIH index

### Evaluation of clinical predictive model for LN

ROC analysis indicated that the multivariate Cox regression model could effectively distinguish the composite endpoint of LN, and the area under the ROC curve (AUC) was 0.82 (95%CI 0.71–0.93) (Fig. 5B). Based on the multivariate Cox regression model, a nomogram was further constructed to facilitate the calculation of the 5-year and 10-year probability of being free from poor prognosis (Fig. 5C).

## Discussion

The formation of crescents in renal tissue usually represents the severity of disease and has important implications for the choice of treatment regimens [13]. However, the significance of crescents and different types of crescents in LN is not fully understood. ISN/RPS 2018 LN pathological classification clarifies the definition of crescent and its different types in the renal biopsy tissue of LN. In this study, based on ISN/RPS 2018 LN pathological classification, we found that cellular/fibrocellular crescent was an important predictor of poor prognosis in LN patients.

Our study showed that patients with crescents in renal tissue had more severe and active disease manifestations, such as higher serum creatinine, lower serum albumin, higher SLEDAI, and higher AI score. Under the condition of consistent baseline demographic characteristics, the long-term prognosis of patients with crescents was worse than that of patients without crescents. This is not completely consistent with the previous studies. Rijnink et al. found that cellular/fibrocellular crescent was associated with the decrease of eGFR in LN patients, but not associated with the progressive deterioration of renal function and ESRD, possibly due to the better response to treatment and slower progression of disease [14]. However, Tao et al. found an independent predictive value for endpoint events when the ratio of cellular/fibrocellular crescents in glomeruli was ≥ 7.39% [15]. We suppose that the reasons for the inconsistent results may include: (1) different grouping criteria for crescents. The classification of crescents in previous LN pathological classification was not clearly defined, resulting in different grouping criteria among studies, which may lead to different results. (2) Influence of treatment regimens. With the application of new immunosuppressants, the treatment for LN have varied in different periods. Different treatment interferes the progress of disease in different degrees and may influence the prognosis of the disease. Our study found that patients with crescents received relatively more aggressive treatment within initial 2 years, which probably associated with improved renal function in patients with crescents in the first 2 years of follow-up, but the treatment regimens included in the study were not significant associated with long-term prognosis of LN.

We further found that the effect of the crescent on the prognosis of LN was influenced by proteinuria. Patients with low level proteinuria (< 3.5 g/24 h) at baseline with crescents had worse long-term prognosis than those without crescents, whereas crescents in patients with high level proteinuria (≥ 3.5 g/24 h) had no significant effect on the long-term prognosis of LN. The difference in histological classification between the two groups is one of the reasons: in patients with low levels of proteinuria, patients with crescents had a higher proportion of class IV, while patients without crescents were dominantly class V. In patients with high levels of proteinuria, class IV was the dominant histological classification in both crescent or non-crescent group. Rezende et al. pointed out that proteinuria mainly reflects podocyte damage in LN, by comparing the podocyte biomarkers, they found that the expressions of synaptopodin (69.2%), WT1 (69.2%), GLEPP1 (53.9%) and nephrin (60.0%) were retained in the pure membranous LN group, while only synaptopodin (2.6%), WT1 (7.7%), GLEPP1 (2.9%) and nephrin (9.4%) were retained in the proliferative LN group. It is speculated that structural podocyte injury occurs in proliferative LN, while only functional podocyte injury occurs in pure membranous LN, which may explain the better prognosis of pure membranous LN [16]. Our study showed that in patients with low levels of proteinuria, serum albumin level in patients with crescents was lower than that without crescents, but in patients with high levels of proteinuria, there was no significant difference between patients with or without crescents. Our previous study found that low levels of proteinuria combined with low levels of serum albumin may indicate more severe extrarenal activity of LN [17]. Therefore, we suppose that in patients with high levels of proteinuria which caused by structural podocyte injury was predominant in both groups with or without crescents; therefore, there was no significant difference in prognosis between the two groups. However, in the patients with low levels of proteinuria, proteinuria in the non-crescent group was predominantly caused by functional podocyte injury, and proteinuria in the crescent group was predominantly caused by structural podocyte injury, and the proportion of patients with extrarenal activity was higher in the crescent group, resulting in a worse long-term prognosis.

Previous studies have shown that a high proportion of SLE patients with low-level proteinuria can be pathologically diagnosed as proliferative or membranous LN [18–20]. Our study found that no difference in median follow-up time between patients with low and high levels of proteinuria at baseline in patients with crescents, suggesting that LN patients with crescents formation have a poor long-term prognosis even they suffered from low level of proteinuria. Therefore, we suggest that renal biopsy should be performed early in LN patients to clarify the renal pathological changes, and to formulate a targeted treatment regimen.

Our study also found that CI of renal tissue was an independent risk factor for poor prognosis of LN, and the higher the CI, the worse the prognosis, which is consistent with previous studies [21, 22]. In addition, the multifactorial Cox regression model showed that positive anti-dsDNA antibody was an independent risk factor for poor prognosis in LN patients. Previous studies have suggested that anti-dsDNA antibody is an independent predictor of proliferative and non-proliferative LN, but not an independent risk factor for poor prognosis in LN patients. Combined detection of anti-dsDNA and anti-C1q antibody plays an important role in evaluating the activity of renal disease and predicting long-term prognosis [23, 24]. In our study, the measurement of anti-dsDNA antibody were only qualitatively detected in blood, while quantitative detection of anti-dsDNA and anti-C1q antibody had not been performed, which may lead to bias in the results. Therefore, the significance of anti-dsDNA antibody and other autoantibodies on the prognosis of LN remains to be further studied.

There are several limitations to this study. First, this was a retrospective, observational study with variation in patients’ conditions. There were differences in the treatment background of the patients, and some patients received treatment, such as immunosuppressants prior to biopsy. Second, the exclusion of some patients from the study cohort due to a follow-up period of < 3 months and loss of some patients during follow-up may have impact on the patients’ outcome. The impact of special types of LN (such as renal vascular lesions and lupus podocytopathy) on clinical features and prognosis was not evaluated in this study, which need to be further studied in the future.

## Conclusions

In summary, our study found that LN patients with cellular/fibrocellular crescents in renal biopsy tissue had more severe and active disease manifestations. The presence of cellular/fibrocellular crescents was a risk factor for poor prognosis in LN patients, and there was a significant interaction between cellular/fibrocellular crescents and proteinuria in predicting poor prognosis. Among patients with low levels of proteinuria at the time of renal biopsy, those with cellular/fibrocellular crescents had a worse long-term prognosis than those without crescents. Meanwhile, we constructed a stable prognosis prediction model of LN to facilitate clinical practice in identifying poor prognosis in LN patients.

## Supplementary Information

**Additional file 1: **Drugs used to maintain remission.

**Additional file 2: **Correlations between crescent and clinical and pathological parameters at the time of renal biopsy.

**Additional file 3: **Treatment regimens of LN patients stratified by proteinuria.

**Additional file 4: **Clinical characteristics of LN patients stratified by proteinuria grouped according to crescent.

**Additional file 5: **Pathological characteristics of LN patients stratified by proteinuria grouped according to crescent.

**Additional file 6: **Treatment regimens of LN patients stratified by proteinuria grouped according to crescent.

## Data Availability

The data sets used and/or analyzed during the current study are available from the corresponding author on reasonable request.

## Abbreviations

ISN/RPS: International Society of Nephrology/Renal Pathology Society
LN: Lupus nephritis
SLE: Systemic lupus erythematosus
ESRD: End-stage renal disease
AI: Activity index
CI: Chronic index
BMI: Body mass index
MBP: Mean arterial pressure
SLEDAI: Systemic lupus erythematosus disease activity index
eGFR: Estimated glomerular filtration rate
WBC: White blood cells
RBC: Red blood cells
ACR: Albumin/creatinine ratio
HR: Hazard ratio
95% CI: 95% Confidence interval
ROC: Receiver operating characteristic
